# The ‘CASTLE’ tumour: An extremely rare presentation of a thyroid malignancy. A case report

**DOI:** 10.1016/j.amsu.2019.06.013

**Published:** 2019-06-30

**Authors:** Diana Mellisa Dualim, Guo Hou Loo, Shahrun Niza Abdullah Suhaimi, Nani Harlina Md Latar, Rohaizak Muhammad, Nordashima Abd Shukor

**Affiliations:** The National University of Malaysia, Jalan Yaacob Latiff, Bandar Tun Razak, Postcode 56000, Selangor, Malaysia

**Keywords:** CASTLE, Thyroid malignancy, Debulking surgery, Chemoradiotherapy

## Abstract

Thyroid carcinoma showing thymic-like differentiation (CASTLE) is a rare malignancy of the thyroid gland, and it accounts for 0.1–0.15% of all thyroid cancers. As the name suggests, it has a histological and immunophenotypic resemblance to thymic carcinoma. Preoperative diagnosis of CASTLE can be difficult as its clinical manifestations, and histological characteristic resembles other aggressive and advanced thyroid carcinomas. It is essential to distinguish CASTLE from other aggressive neoplasms as the former has a more favourable prognosis. Immunohistochemical staining with CD5 can help to differentiate thyroid CASTLE from other aggressive thyroid neoplasms.

Due to the rarity of this disease, there is no clear definitive treatment strategy. Surgical resection of CASTLE is usually attempted initially. Nodal involvement and extrathyroidal extension are shown to be the main prognostic factors that influenced the survival of patients. Therefore, complete resection of the tumour is vital to reduce local recurrence rates and to improve the chance of long-term survival. Radiotherapy (RT) for CASTLE is an effective treatment. Curative surgery followed by adjuvant RT should be considered in cases with extrathyroidal extension and nodal metastases. With RT, shrinkage of the tumour and reduction of local recurrence rate is possible.

With that in mind, we present a case of CASTLE who presented with airway compression symptoms three years after thyroid surgery. He subsequently underwent tumour debulking surgery and a tracheostomy. The patient refused adjuvant chemoradiotherapy, and during our serial follow-up, he is well and symptom-free.

## Highlights

• Thyroid carcinoma showing thymic-like differentiation (CASTLE) is a rare malignancy of the thyroid gland.
• Diagnosis of CASTLE can be difficult as it resembles other aggressive and advanced thyroid carcinomas.
• Immunohistochemical staining with CD5 can help to differentiate thyroid CASTLE from other aggressive thyroid neoplasms.
• Complete surgical resection is vital to reduce local recurrence rates and to improve the chance of long-term survival.
• Adjuvant radiotherapy should be considered in cases with extrathyroidal extension and nodal metastases.

## 1 Introduction

Thyroid carcinoma showing thymic-like differentiation (CASTLE) is a rare malignancy of the thyroid gland, and it accounts for 0.1%–0.15% of all thyroid cancers [1,2]. As its name suggests, CASTLE has a histological and immunophenotypic resemblance to thymic carcinoma [2]. Preoperative diagnosis of CASTLE can be difficult because its clinical manifestations and histological characteristics resemble those of other aggressive and advanced thyroid carcinomas [2,3]. It is imperative to distinguish CASTLE from other aggressive neoplasms because the former has a more favourable prognosis. We present a case of CASTLE who presented with airway compression symptoms three years after thyroid surgery. We discuss issues related to the difficulty of diagnosing and managing the tumour. This case is reported in line with the SCARE criteria [4].

## 2 Case presentation

A 58-year-old man presented to us three years ago with a history of progressive anterior neck swelling and hoarseness of voice for the past one month. On clinical examination, there was palpable left anterior neck swelling with cervical lymphadenopathy. A core needle biopsy of the lesion revealed invasive poorly differentiated carcinoma. He defaulted our follow-up and opted for a left hemithyroidectomy at another institution. The histopathological report was intrathyroid thymic carcinoma. He refused adjuvant radiotherapy despite being counselled and subsequently defaulted follow-up.

**Fig. 1:**
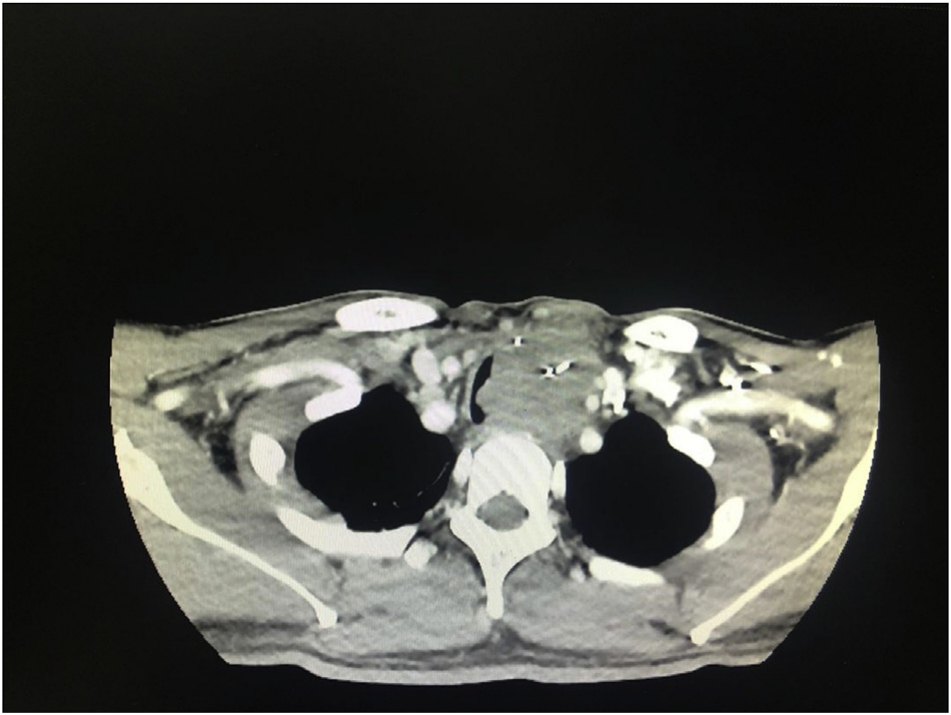
Axial view of the CECT neck showing a lobulated hypodense soft tissue mass measuring 4.3 cm × 3.9 cm ×5.2cm. It occupies the left thyroid bed from C7/T1 to T2/T3 vertebral level.

**Fig. 2:**
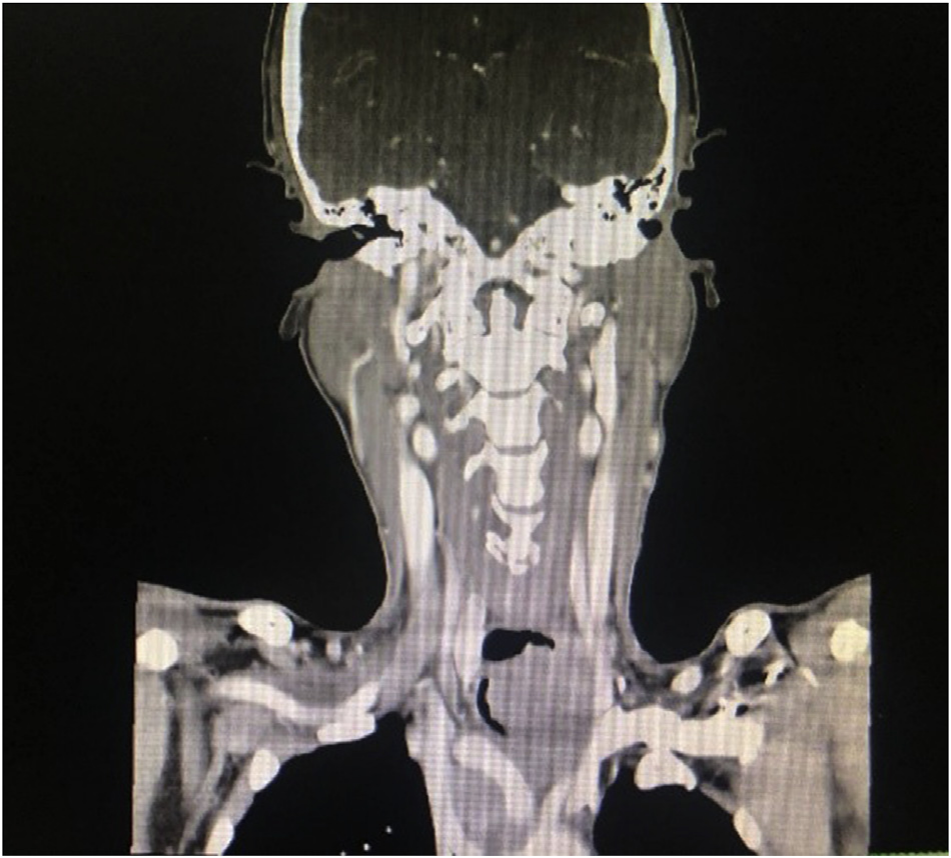
Coronal view of the CECT neck showing the lobulated hypodense soft tissue mass, causing more than 80% of tracheal luminal narrowing.

Recently, the patient presented to us again with progressive hoarseness of voice and intermittent haemoptysis. He had shortness of breath, especially when lying flat, but no difficulty in swallowing. On clinical examination, there was a left supraclavicular mass measuring 2 × 2 cm. The mass was immobile and hard in consistency. The anterior aspect of the neck showed an irregular hard mass measuring 3 × 4 cm. An indirect laryngoscopy examination revealed a left vocal cord palsy, likely from his previous presentation. An intraluminal mass was seen over the posterior tracheal wall, occupying a third of the tracheal lumen. We proceeded with a contrasted computed tomography (CECT) of the neck and thorax, which revealed a lobulated hypodense soft tissue mass measuring 4.3 × 3.9 × 5.2 cm. It occupied the left thyroid bed from the C7/T1 to the T2/T3 vertebral level, with left retrosternal extension and tracheal deviation to the right. The soft tissue mass had displaced the left common carotid artery and internal jugular vein laterally. As the CECT showed (Fig. 1, Fig. 2), the soft tissue mass had caused more than 80% of tracheal luminal narrowing. At retrosternal, multiple matted lymph nodes were seen.

**Fig. 3:**
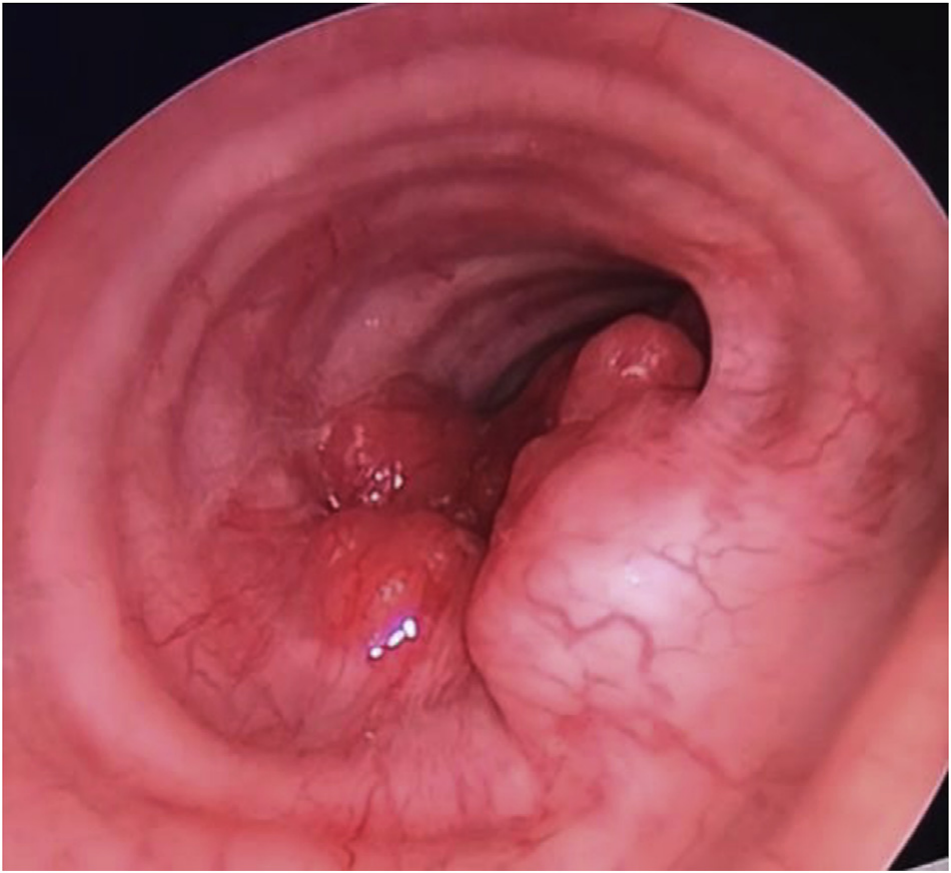
Fibreoptic endoscopic view of trachea showing soft tissue mass measuring 3cm in length at the posterior tracheal wall.

Image-guided fine needle aspiration cytology (FNAC) of the soft tissue mass at the left thyroid bed showed malignant cells suggestive of intrathyroid thymic carcinoma (CASTLE). After a multidisciplinary meeting, we planned for curative resection and tracheostomy. Intraoperative tracheoscopic examination showed a soft tissue mass measuring 3 cm in length at the posterior tracheal wall, 2.1 cm from the vocal cord (Fig. 3). The mass was debrided to achieve an adequate diameter of tracheal lumen distal to the tracheostomy insertion. The left supraclavicular solid tumour densely adhered to the left great vessels. Due to a high risk of injury to the great vessels, we proceeded with tumour debulking only.

**Fig. 4:**
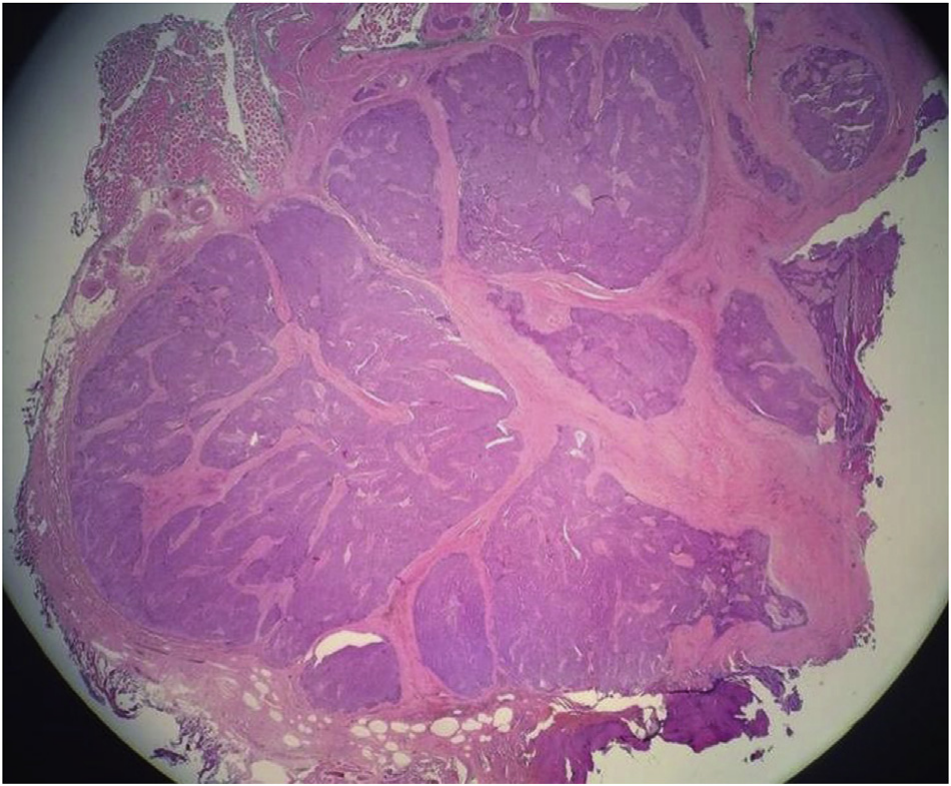
Microscopic examination of the sections from left thyroid soft tissue mass at 1.25× and 20× magnification showing the unencapsulated tumour that displayed lobular architecture. The tumour lobules were composed of sheets of neoplastic polygonal cells rimmed by a variable amount of mature lymphocytes. The neoplastic cells were mildly pleomorphic.

**Fig. 5:**
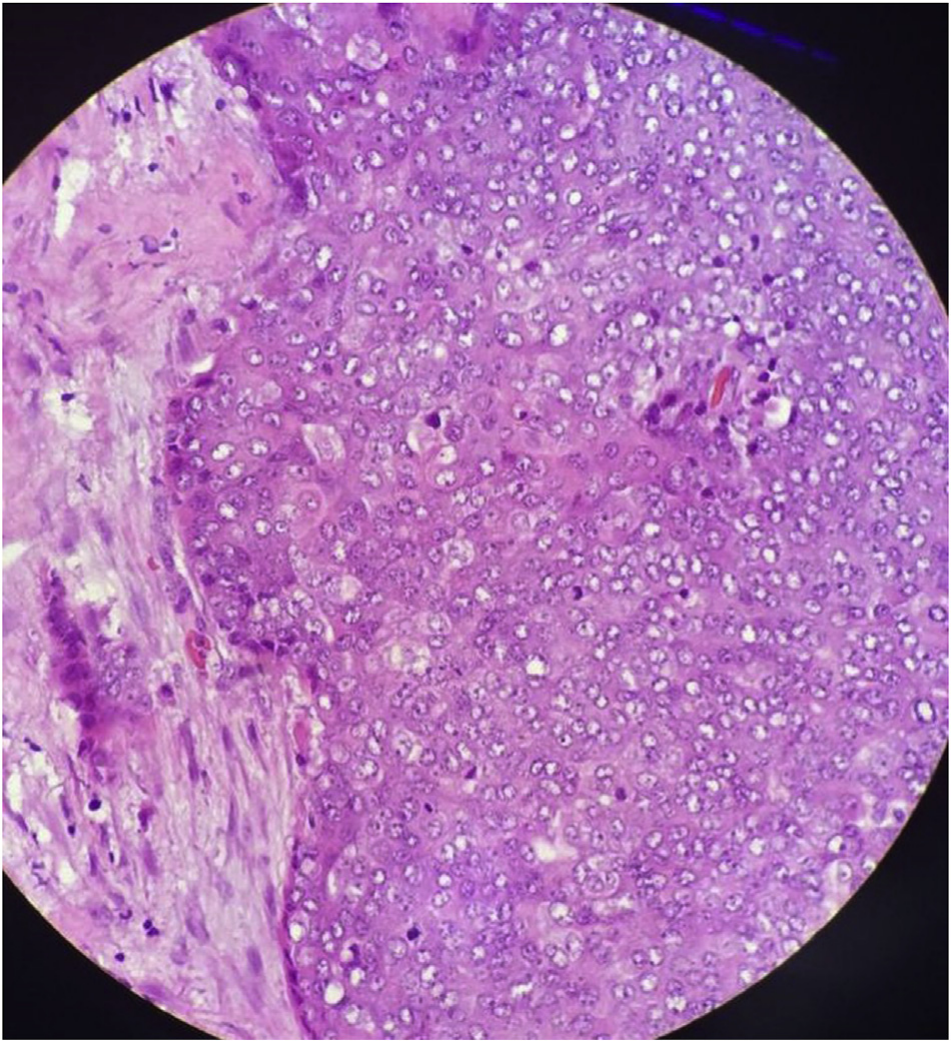

**Fig. 6:**
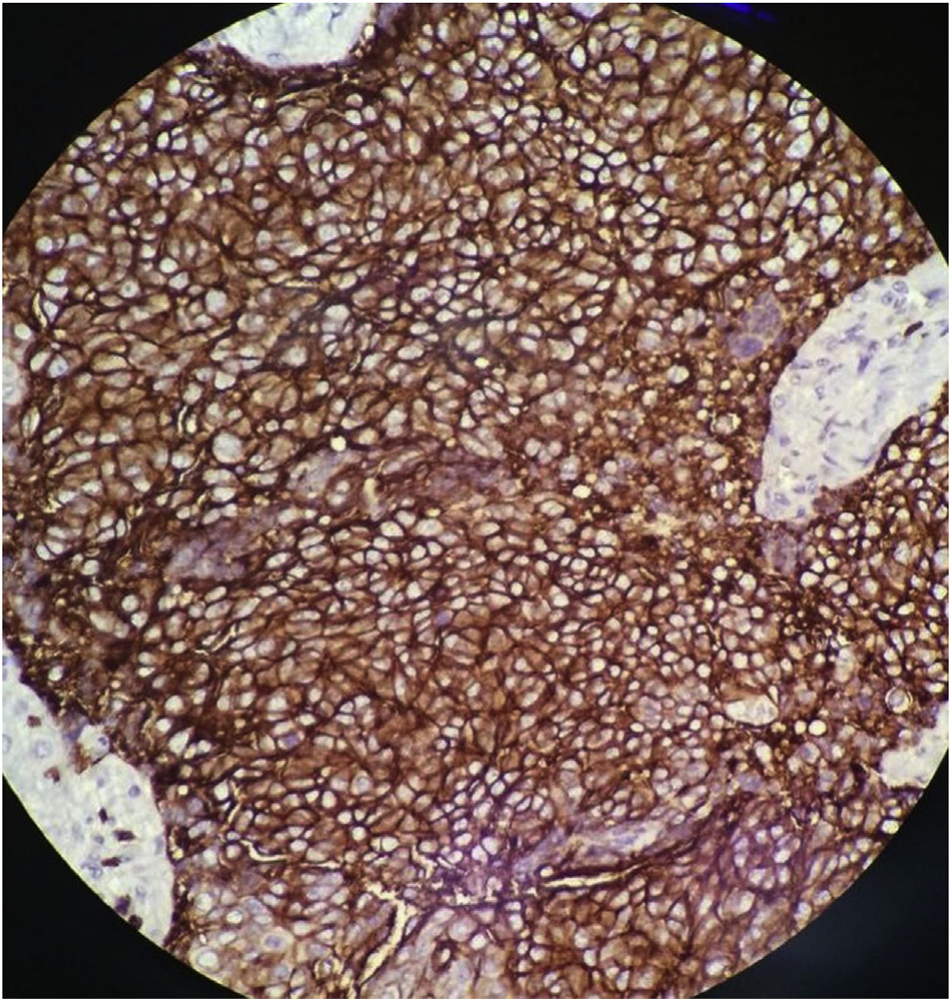
Microscopic examination of immunohistochemical studies at 20× magnification showing immunoreactivity for CD5 (membranous pattern) and CD117, respectively.

**Fig. 7:**
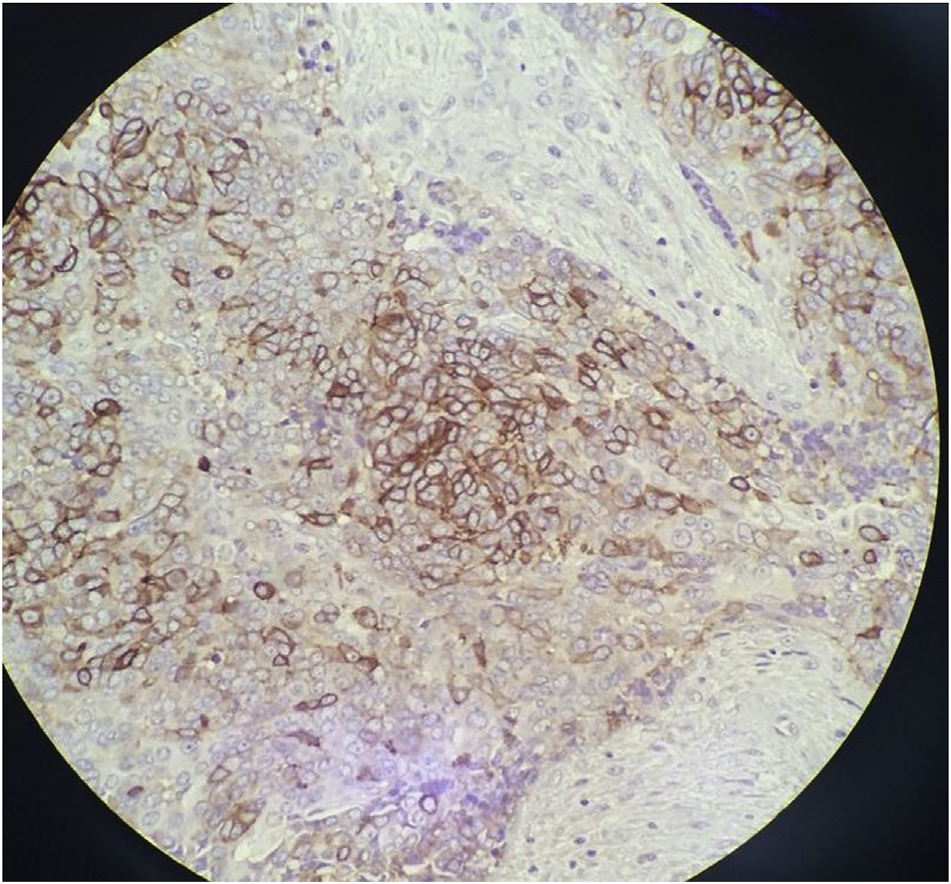

The histopathology report showed an unencapsulated tumour that displayed lobular architecture. The tumour lobules were composed of sheets of neoplastic polygonal cells rimmed by a variable amount of mature lymphocytes. The neoplastic cells were mildly pleomorphic, and lymphovascular invasion was present. Skeletal muscle bundles attached at the periphery showed focal tumour cell infiltration (Fig. 4, Fig. 5). Immunohistochemical studies were immunoreactive for CD5 (membranous pattern) and CD117 but negative for thyroglobulin and TTF1 (Fig. 6, Fig. 7). The proliferative index, Ki67, was approximately 20%.

Postoperative care was unremarkable, and no immediate complications were noted. The patient was counselled for adjuvant chemoradiotherapy, but he was not amenable to this course of action. He is currently under regular follow-up with no disease progression six months after the operation.

## 3 Discussion

CASTLE is a rare entity of malignant thyroid neoplasm that accounts for 0.1%–0.15% of all thyroid cancers [1,2]. It was discovered by Miyauchi et al. [5] in 1985 as ‘intrathyroidal epithelial thymoma’. In 1991, Chan and Rosai classified these tumours into four types: ectopic hamartomatous thymoma, ectopic cervical thymoma, spindle epithelial tumours with thymic-like differentiation (SETTLE), and CASTLE [5]. The first two types are considered benign, because they share histological features with intrathymic thymomas, whereas SETTLE and CASTLE exhibit malignant characteristics. In 2004, the World Health Organization designated CASTLE as an independent clinicopathological entity among thyroid carcinomas. It has a histological and immunophenotypic resemblance to thymic carcinoma [2], and it likely originates from an ectopic thymus or branchial pouch remnants [2,6].

Preoperative diagnosis of CASTLE can be difficult because its clinical manifestations and histological characteristics resemble other aggressive and advanced thyroid carcinomas. It is imperative to distinguish other aggressive thyroid neoplasms from CASTLE because the later has a more indolent clinical course and a better prognosis [3,6].

CASTLE usually affects patients in their fourth and fifth decades of life and has a slight female predominance [9]. CASTLE commonly presents as a slow-growing, painless cervical mass [6]. However, the clinical manifestations may vary and are not specific to the disease because the tumours may also invade adjacent soft tissues and regional lymph nodes [2,9]. CASTLE may also present with swallowing difficulties resulting from the mass and vocal hoarseness due to recurrent laryngeal nerve involvement [8,9]. Other symptoms include haemoptysis and dyspnoea [9]. Although uncommon, there are reports of CASTLE metastases to the brain, liver, and lungs [5].

Imaging modalities such as ultrasonography of the neck, CECT head and neck, and magnetic resonance imaging may guide the diagnosis of CASTLE but are usually nonspecific [10]. In a non-enhanced computed tomography of the neck, CASTLE often displays a soft tissue density with an ill-defined border without calcification [6,9].

FNAC plays a crucial role in the diagnosis of differentiated thyroid carcinoma, especially papillary carcinoma, which accounts for 90% of thyroid cancers. However, cytology has its limitations, and it is challenging to distinguish CASTLE from poorly differentiated carcinoma, squamous cell carcinoma, or anaplastic thyroid carcinoma [2,3]. A core needle biopsy can obtain tissue samples of sufficient size to perform immunohistochemical staining. Immunohistochemical studies can differentiate CASTLE from these other aggressive thyroid neoplasms [6]. CASTLE is immunohistochemically positive for CD5, p63, and cytokeratin and negatively stains for thyroglobulin, thyroid transcription factor-1 (TTF1), and calcitonin [6,9]. CD5 is explicitly valuable because it can help distinguish CASTLE from other tumours of the thyroid gland or the upper aerodigestive tract [11].

CASTLE is usually characterised by its indolent biological behaviour, which has a relatively favourable prognosis [6]. Due to the rarity of this disease, there is no definitive treatment strategy. Surgical resection of CASTLE is usually attempted initially. Thyroidectomy with neck dissection should always be performed [6]. Central compartment dissection is necessary for all cases of CASTLE, especially in patients with lymph nodes that are clinically not palpable. Cases of suspected or proven lateral compartment involvement should receive selective neck dissection or modified neck dissection [2,6]. Previous reports have suggested the need to perform curative surgery with neck dissection to achieve favourable outcomes with a locoregional recurrence rate of 14% and 5- and 10-year cause-specific survival rates of 90% and 82%, respectively [2]. The lymph node metastasis rates are about one-third, and there are reported cases of progression to metastatic disease [7]. The 5- and 10-year cause-specific survival rates for CASTLE are 90% and 82%, respectively [8]. The incidence of extrathyroidal extension is 50%–60% and was shown to be one of the prognostic factors besides nodal involvement that influenced the survival of patients [9]. Therefore, complete resection of the tumour and involved structures is vital to reducing local recurrence rates and improving the likelihood of long-term survival.

In cases of CASTLE with tracheal invasion, there are reported data of successful airway resection with preservation of larynx. Surgical resection is feasible because the invasion is confined mostly to the lower part of the cervical trachea. This is because CASTLE tends to arise from the lower pole of the thyroid gland [2,9]. Surgery alone without adjuvant radio- or chemotherapy is sufficient for cases of CASTLE without nodal involvement or extrathyroidal extension [2,9].

Radiotherapy can be used in CASTLE because it has been reported to be radiosensitive [1,2]. Surgery combined with adjuvant radiotherapy has been shown to improve the survival of CASTLE patients [10]. A beneficial effect was seen in cases without lymph node metastasis and tumour extension [12]. The locoregional recurrence was higher in patients who did not receive radiotherapy [12]. Another modality available for CASTLE treatment is chemotherapy; however, studies have failed to show improved patient survival [2,10].

## 4 Conclusion

CASTLE is a rare malignancy of the thyroid gland. Preoperative diagnosis of CASTLE can be difficult because its clinical manifestations and histological characteristics resemble those of other aggressive and advanced thyroid carcinomas. The management of this tumour is challenging due to its rarity and the limited evidence available. Extrathyroidal extension and nodal metastasis are the main prognostic factors for determining the risk of local recurrence and the survival of CASTLE patients.

## Declaration of conflict of interest

None.

## Source of funding

None.

## Consent

Written informed consent was obtained from the patient for publication of this case report and accompanying images. A copy of the written consent is available for review by the Editor-in-Chief of this journal on request.

## Ethical approval

The National University of Malaysia Research Ethics Committee has exempted the need for an ethical approval for any case report being written/published.

## Sources of funding

No source of funding.

## Author contribution

Study concepts: Shahrun Niza Abdullah Suhaimi, Nani Harlina Md Latar, Rohaizak Muhammad, Study design: Shahrun Niza Abdullah Suhaimi, Nani Harlina Md Latar, Rohaizak Muhammad.

Data acquisition: Diana Mellisa Dualim, Loo Guo Hou, Quality control of data and algorithms: Shahrun Niza Abdullah Suhaimi, Nani Harlina Md Latar, Rohaizak Muhammad.

Data analysis and interpretation: Diana Mellisa Dualim, Loo Guo Hou.

Statistical analysis: Not applicable.

Manuscript preparation: Loo Guo Hou, Diana Mellisa Dualim.

Manuscript editing: Loo Guo Hou, Shahrun Niza Abdullah Suhaimi.

Manuscript review: Shahrun Niza Abdullah Suhaimi, Rohaizak Muhammad.

## Conflicts of interest

No conflict of interests.

## Trial registry number – ISRCTN

Not applicable.

## Guarantor

Shahrun Niza Abdullah Suhaimi.

## Research registration Unique Identifying number (UIN)

Name of the registry: National Medical Research Register (NMRR) Malaysia.

Unique Identifying number or registration ID: NMRR-19-1128-48418.

## Provenance and peer review

Not commissioned, externally peer reviewed.
